# Fabrication of Fe_3_O_4_@mSiO_2_ Core-Shell Composite Nanoparticles for Drug Delivery Applications

**DOI:** 10.1186/s11671-015-0920-5

**Published:** 2015-05-13

**Authors:** Sergio I Uribe Madrid, Umapada Pal, Young Soo Kang, Junghoon Kim, Hyungjin Kwon, Jungho Kim

**Affiliations:** Instituto de Física, Universidad Autónoma de Puebla, Apdo. Postal J-48, Puebla, 72570 Mexico; Department of Chemistry, Korea Center for Artificial Photosynthesis, Sogang University, 35, Baekbeom-ro, Mapo-gu, Seoul 121-742 Republic of Korea; Department of Life Science, Laboratory of Molecular and Cellular Biology, Sogang University, 35, Baekbeom-ro, Mapo-gu, Seoul 121-742 Republic of Korea

**Keywords:** Magnetite, Core-shell, Porous composite nanoparticle

## Abstract

We report the synthesis of Fe_3_O_4_@mSiO_2_ nanostructures of different meso-silica (mSiO_2_) shell thickness, their biocompatibility and behaviors for loading and release of a model drug ibuprofen. The composite nanostructures have superparamagnetic magnetite cores of 208 nm average size and meso-silica shells of 15 to 40 nm thickness. A modified Stöber method was used to grow the meso-silica shells over the hydrothermally grown monodispersed magnetite particles. The composite nanoparticles show very promising drug holding and releasing behaviors, which depend on the thickness of meso-silica shell. The biocompatibility of the meso-silica-coated and uncoated magnetite nanoparticles was tested through cytotoxicity assay on breast cancer (MCF-7), ovarian cancer (SKOV3), normal human lung fibroblasts MRC-5, and IMR-90 cells. The high drug holding capacity and reasonable biocompatibility of the nanostructures make them ideal agents for targeted drug delivery applications in human body.

**Electronic supplementary material:**

The online version of this article (doi:10.1186/s11671-015-0920-5) contains supplementary material, which is available to authorized users.

## Background

In recent decades, nanotechnology has made great strides in the design of new materials, with distinct characteristics and functionalities suitable for applications in specific areas such as in biomedicine [1], targeted drug delivery [2–4], and photodegradation of environmental pollutants [5–7]. For targeted drug delivery applications, one of the main challenges is to develop nanostructures that can be loaded with special drug and can be transported to certain specific location of the body in a simple manner [8]. In this regard, magnetite has been the most studied material, especially as T2 contrast agent in MRI [9] due to its biocompatibility and adequate magnetic properties. When the magnetite is synthesized at nanoscale, its magnetic properties change from ferromagnetic to superparamagnetic, extending its application to biomedicine [10–12]. However, when magnetite nanoparticles are naked, they get agglomerated due to high magnetic interaction and high surface area. Moreover, in biological media, they can be easily oxidized to form other phases. On the other hand, reticuloendothelial system (RES) of human body takes up bigger (>300 nm) magnetite nanoparticles from circulatory blood more quickly than smaller sizes [13, 14]. Therefore, it is necessary to functionalize magnetite nanoparticles modifying their surface characteristics or cover them properly by another biocompatible material to avoid these disadvantages [15–18]. Frequently, mesoporous silica (meso-silica) has been utilized as vehicle for the delivery of special drugs [19–21] due its good biocompatibility in human body and high specific surface area (high drug loading capacity). However, the problem arises when we need to bring the drug to a specific site using meso-silica as vehicle. Therefore, it is necessary to functionalize such mesostructures in a special way to achieve the objectives [22].

Magnetite nanoparticles covered with meso-silica shells (Fe_3_O_4_@mSiO_2_) have been seen to be the most promising material, fulfilling most of the abovementioned criteria for applying them as vehicles for delivering special drugs at specific sites of human body. While Xu et al. [23] have developed Fe_3_O_4_@nSiO_2_@mSiO_2_ composite nanoparticles with average size of about 400 nm, with good (about 95 % in 85 hours) drug (ibuprofen)-releasing properties, Xu and collaborators [24] fabricated hollow Fe_3_O_4_@SiO_2_ spheres of about 900 nm average size with high drug (aspirin) loading and sustained drug-releasing capacity. However, the sizes of their nanoparticles are not perfectly suitable for targeted drug delivery, as nanoparticles below 300 nm are desired for this application [13, 25]. In this work, we report the synthesis of magnetite core meso-silica shell composite nanoparticles of different shell thicknesses using hydrothermal and sol–gel techniques. The structure, morphology, and texture characteristics of the nanostructures have been studied using SEM, TEM, and nitrogen adsorption–desorption techniques. The drug-holding and -releasing capacity of the nanostructures have been studied using ibuprofen as a model drug. Finally, we tested the cytotoxicity of nanostructures to check their biocompatibility with the human body.

## Methods

### Chemicals and Solvents

Ferric chloride hexahydrate (FeCl_3_·6H_2_O, 97 %), anhydrous sodium acetate (NaAc) (CH_3_COONa, 99.9 %), tetraethylorthosilicate (TEOS), cetyltrimethylammonium bromide (CTAB), glacial acetic acid, and ibuprofen were obtained from Sigma-Aldrich, St. Louis, MO, USA. Ethylene glycol (EG, C_2_H_6_O_2_, 99.7 %) was purchased from J. T. Baker, Naucalpan, Edo. de México, Mexico. E-pure deionized (DI) water (ρ > 18.2 Ω·cm) was used as solvent for washing. All the chemicals were of analytical grade and used without further purification.

### Synthesis of Magnetite (Fe_3_O_4_) Nanoparticles

Magnetite nanoparticles were synthesized through EG mediated solvothermal process following the procedure we reported earlier [26]. In a typical synthesis process, first a 0.6-M solution of FeCl_3_ in EG was prepared by dissolving 1.6211 g of FeCl_3_·6H_2_O in 10 mL of EG. The solution was added to 40 mL of EG in a three-necked round bottom flask under Ar atmosphere under magnetic agitation. After 30 min of stirring, 10 mL of 1.2 M sodium acetate solution in EG was added to the previous mixture under vigorous stirring. The stirring process was continued for another 3 hours, and then the mixture was transferred to a Teflon-lined stainless steel autoclave and heated at 190 °C for 24 hours. Finally, the autoclave was cooled down to room temperature, and the product was magnetically separated, washed with water and ethanol several times, and dried at 65 °C for 12 hours.

### Synthesis of Fe_3_O_4_@mSiO_2_ Nanostructures

For fabricating meso-silica covers over the prefabricated magnetite nanoparticles, we used a modified Stöber method [27] very similar to the method we reported earlier [26]. About 10 mg of prefabricated magnetite nanoparticles were dispersed in 50 mL of ethanol under ultrasonic agitation. After that, the nanoparticles were magnetically separated and redispersed in a 200-mL solution of water/ethanol (1:4, *v*/*v*). Five milliliters of NH_4_OH (28 wt.%) was added to the previous mixtures. After about 30 min of stirring, 150 mg (0.41 mmol) of CTAB was added into the solution, and the stirring was continued for further 30 min. Then, a desired amount of TEOS (0.05, 0.07, or 0.10 mL) was added to the mixture dropwise. After 24 hours of mechanical stirring at room temperature, the nanostructures were magnetically separated, washed with ethanol and water, and redispersed in 100 mL ethanol/acetic acid (95:5, *v*/*v*) solution to remove CTAB from the sample. After about 30 min of stirring, the precipitate was separated magnetically, washed with water and ethanol several times, and then dried in an oven at 80 °C for 24 hours. The Fe_3_O_4_@mSiO_2_ composite nanoparticles synthesized with 0.05 (0.22 mmol), 0.07 (0.31 mmol), and 0.10 mL (0.45 mmol) of TEOS were named as samples SG-1, SG-2, and SG-3, respectively.

### Characterization

Field emission high-resolution scanning electron microscopy (FE-HRSEM; Zeiss Auriga 3916, Carl Zeiss AG, Oberkochen, Germany) and transmission electron microscopy (TEM, JEOL-JEM 211F operated at 200 keV, JEOL Ltd., Tokyo, Japan) were used to determine the size and morphology of the synthesized nanostructures. To determine the specific surface area of the samples, their N_2_ adsorption–desorption isotherms at 77 K were recorded using Autosorb-1 Quantachrome Instrument sorptometer (Quantachrome Instruments, Boynton Beach, FL, USA). A UV–vis–NIR spectrophotometer (Cary 7000, Agilent Technologies, Santa Clara, CA, USA) was used to monitor the ibuprofen loading and releasing behavior of the samples. A PPMS DynaCool (Quantum Design, San Diego, CA, USA) system was used to measure the room temperature magnetization curves (*M* vs. *H*) of the magnetite and meso-silica covered magnetite samples.

### Drug Loading in the Composite Nanoparticles

To test the drug-loading capacity of the Fe_3_O_4_@mSiO_2_ nanostructures, about 30 mg of each of the Fe_3_O_4_@mSiO_2_ samples (SG-1, SG-2, and SG-3) was separately added into 10 mL of ibuprofen/hexane solution (30 mg/mL), and the solution was stirred for 24 hours in a sealed vessel to prevent solution evaporation. After that, the sample was separated magnetically, washed carefully with hexane, and dried in vacuum at 60 °C for 24 hours. The ibuprofen-loaded samples were named as ibu/SG-1, ibu/SG-2, and ibu/SG-3.

### In Vitro Drug-Release From the Nanostructures

The prepared ibu/SGx (*x* = 1–3) samples were immersed in 60 mL of simulated body fluid (SBF, pH = 7.4) under slow stirring at 37 °C. The SBF was prepared following the procedure reported by Chavan et al. [28]. In brief, about 7.996 g of NaCl, 0.350 g of NaHCO_3_, 0.224 g of KCl, 0.228 g of K_2_HPO_4_·3H_2_O, 0.305 g of MgCl_2_·6H_2_O, 0.278 g of CaCl_2_, 0.071 g of Na_2_SO_4_, and 6.057 g of (CH_2_OH)_3_CNH_2_ were dissolved in 500 mL of deionized (DI) water. Then, about 40 mL of 1 M HCl was added to it. The total volume of the mixture was adjusted to 1 L by adding DI water further to the previous mixture, obtaining a solution of pH 7.4. The ratio of SBF to adsorbed ibu was kept at 1 mL/mg in the mixture. At selected time intervals, aliquots (0.5 mL) were removed from the mixture solution, replacing by an equal volume (0.5 mL) of fresh SBF. The amount of ibuprofen released was estimated by monitoring the 263-nm absorption band of ibuprofen in the UV–vis absorption spectra of the aliquots.

### Cell Culture

Human breast cancer cells (cell line MCF7) were cultured in Dulbecco’s modified Eagle’s medium (DMEM) (Gibco, Waltham, MA, USA) supplemented with 10 % fetal bovine serum (Invitrogen, Carlsbad, CA, USA) and 1× antibiotics (Invitrogen 15240-062, Gibco, containing 100 units penicillin and 100 μg of streptomycin per mL). Human ovarian cancer cells (cell line SKOV3) were cultured in Roswell Park Memorial Institute medium (RPMI medium-1640) (HyClone, Logan, UT, USA) supplemented with 10 % fetal bovine serum and 1× antibiotics. Normal human lung fibroblast IMR-90 and MRC-5 cells were cultured in Eagle’s Minimum Essential Medium (EMEM) (Gibco, Waltham, MA, USA) supplemented with 10 % fetal bovine serum and 1× antibiotics. The cultured cells were maintained at 37 °C under 5 % CO_2_ in a humid atmosphere.

### Cytotoxicity Assay

For this purpose, 2 × 10^4^ MCF-4 cells, 1 × 10^4^ SKOV3 cells, 2 × 10^4^ MRC-5 cells, and 2 × 10^4^ IMR-90 cells were plated onto 35 mm dishes (SPL Life Science, Pocheon, Korea) and cultured for 2 days. After 2 days, 2 mL of fresh media were individually replaced with 20 μg/mL of M-1 (uncoated magnetite nanoparticles), SG-1, SG-2, or SG-3 samples. The cell viability tests were performed in triplicates for each of the biological cells. The test cells were monitored using an inverted phase-contrast microscope after 2 days exposure to the magnetic samples. The number of viable cells was determined using ADAM automatic cell counter (Digital Bio, Seoul, Korea) attached with a Nikon ECLIPSE TE300, Tokyo, Japan, microscope at ×100 magnification.

## Results and Discussion

**Fig. 1:**
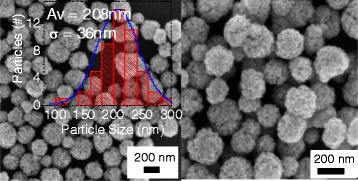
Typical SEM images of Fe_3_O_4_ nanoclusters (M-1) in two magnifications. Their size distribution histogram is presented as *inset*

Figure 1 shows the typical SEM images of the magnetite sample (M-1) synthesized by hydrothermal method. As we can see, well-dispersed spherical nanoclusters of 100 to 300 nm diameters with an average size of 208 nm were formed in the sample (Fig. 1 and Additional file 1: Figure S3). It is very clear that the clusters are formed through the agglomeration of smaller (~20 nm) particles (primary particles). XRD analysis (Additional file 1: Figure S1) of the sample revealed their spinel inverse magnetite phase, with average grain size of about 20 nm (using Scherrer relation).

**Fig. 2:**
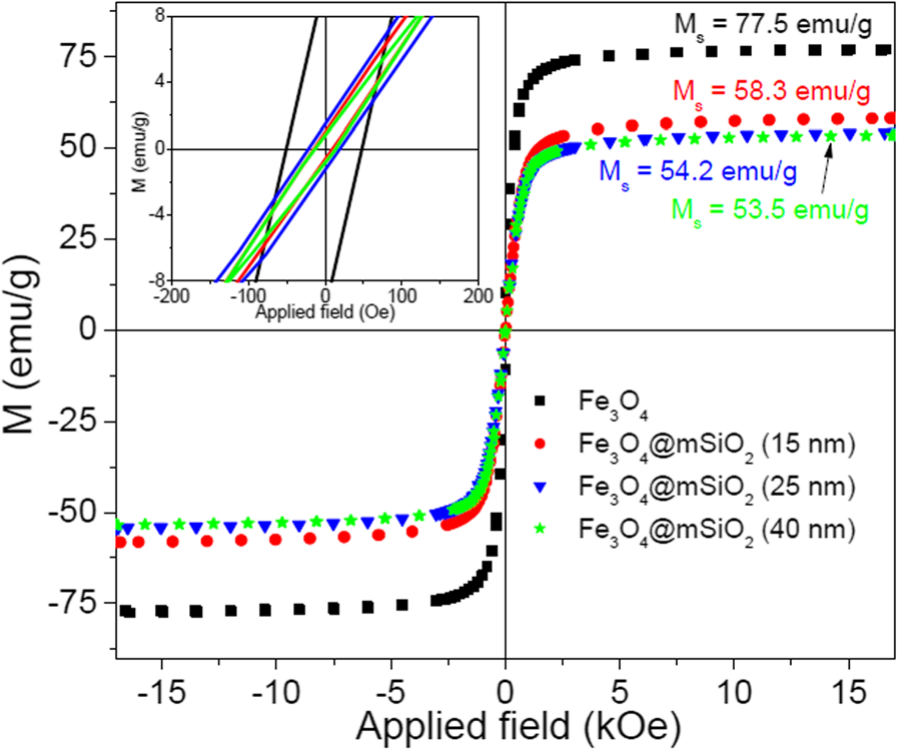
Room temperature magnetization curves for the naked and meso-silica-covered magnetite clusters

The magnetization curve of the sample (M-1) presented in Fig. 2 shows its superparamagnetic behavior with high saturation magnetization (77.5 emu/g). This value is higher than the *M*_s_ values reported by several research groups for the MNPs prepared by different synthesis techniques [29–31]. Such a high *M*_s_ value makes our superparamagnetic nanoparticles suitable for drug delivery applications. It should be noted that the *M*_s_ for bulk magnetite is as high as 92 emu/g [32]. Although the *M*_s_ value of the particles decreases about 25 % due to meso-silica coating, apparently the thickness of the meso-silica layer has no significant effect. On the other hand, meso-silica layer over the magnetite nanoparticles reduces their coercivity (inset of Fig. 2). The high saturation magnetization of the meso-silica coated magnetite particles suggests their suitability for targeted drug delivery applications.

**Fig. 3:**
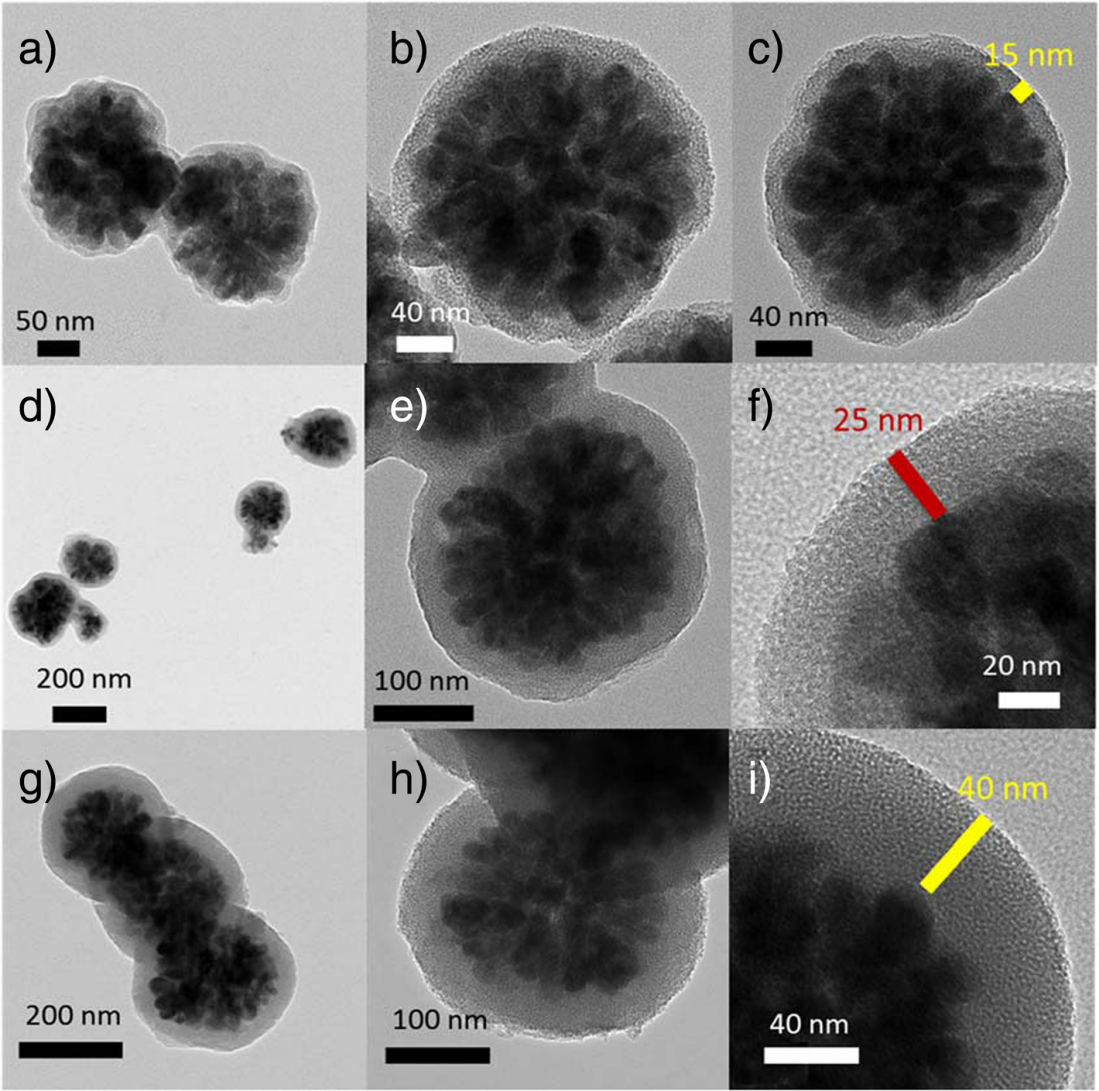
Typical TEM images of the **a–c** SG-1, **d–f** SG-2, and **g–i** SG-3 samples, showing the formation of Fe_3_O_4_@mSiO_2_ core-shell structures with shell thickness 15, 25, and 40 nm, respectively

Using a modified Stöber method, mesoporous silica layers were formed over the magnetite nanoclusters to obtain Fe_3_O_4_@mSiO_2_ core-shell structures. Formation of Fe_3_O_4_@mSiO_2_ nanostructures with silica shells of 15, 25, and 40 nm average thicknesses can be seen in Fig. 3, for the samples prepared using 0.05, 0.07, and 0.10 mL of TEOS in the reaction mixtures, respectively. To make the silica shell mesoporous, we used the cationic surfactant CTAB as a polymer template during its growth, which could be removed after the formation of silica layer by rinsing in ethanol/acetic acid solution (95:5, *v*/*v*). The complete removal of CTAB from the composite nanostructures during this prolonged rinsing process has been demonstrated in their FT-IR spectra (Additional file 1: Figure S2).

**Fig. 4:**
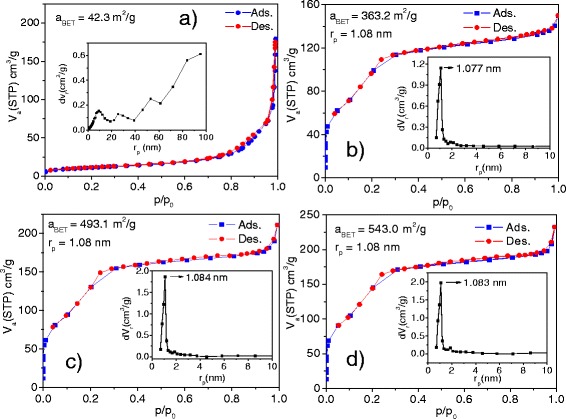
N_2_ adsorption–desorption isotherms of **a** M-1 (Fe_3_O_4_), **b** SG-1, **c** SG-2, and **d** SG-3 samples and their pore size distribution (*insets*)

The surface area and texture properties of the silica covered and naked magnetite particles were studied by recording their N_2_ adsorption–desorption isotherms at 77 K and are presented in Fig. 4. While the adsorption–desorption isotherm of the naked magnetite clusters (Fig. 4a) revealed characteristics of both type II and type IV porous materials (IUPAC classification) [33] due to inhomogeneous pore distribution, all the composite samples (SG-1, SG-2, and SG-3) revealed the characteristics of type IV mesoporous material, indicating their layered mesoporous structures. As the naked magnetite clusters consist of interconnected primary particles of about 20 nm size (see Additional file 1: Figure S3) formed by agglomeration without any order, the mixed macro- and mesoporous nature of the sample is understandable [34]. On the other hand, the mesoporous nature of the composite particles comes from the columnar outer silica layers formed by replicating the lamellar structure of the polymer template CTAB, formed over the magnetite clusters. From the pore size distribution presented as inset of the Fig. 4b–d, we can see that the pore size in all the silica-covered magnetite samples is constant (≈2.16 nm). The BET estimated specific surface area of the M-1, SG-1, SG-2, and SG-3 samples were of 42.3, 363.2, 493.1, and 543.0 m^2^/g, respectively. Therefore, the specific surface area of the composite nanostructures could be controlled by controlling the thickness of the meso-silica layer.

**Fig. 5:**
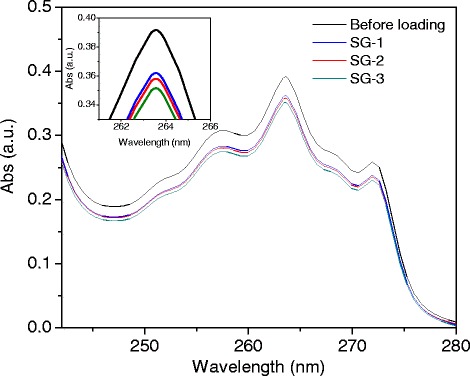
UV absorption spectra of the ibuprofen–hexane solution before (*black line*) and after loading SG-1, SG2, and SG-3 samples. Loading of ibuprofen in the porous structures is clear from the reduction of absorption intensity (*inset*). The *inset* shows the magnified portion of the spectra around 263-nm absorption band of ibuprofen

Figure 5 shows the UV–vis absorption spectra of ibuprofen/hexane solution (30 mg/mL) before (black line) and after loading the SG-1, SG-2, and SG-3 samples (red, green, and blue line, respectively). As we can see, the intensity of the typical 263-nm absorption band decreases with the increase of shell thickness of the composite particles, indicating a higher loading of ibuprofen for the particles with higher meso-silica thickness. Using a pre-calibrated absorbance curve of ibuprofen–hexane solution (Additional file 1: Figure S4), the amount of drug loaded in each of the composite samples could be determined. The estimated amounts of ibuprofen loaded in the samples after 24 hours of impregnation under hexane solution (under agitation at 25 °C) were 678, 828, and 954 mg_ibu_/mg_sample_, respectively (Table 1). Therefore, on increasing the thickness of meso-silica layer over magnetite clusters, a higher amount of drug could be loaded. The drug-loading capacity of our composite nanoparticles is comparable to the drug-loading capacity of pure mesoporous silica nanoparticles reported by Mei et al. [21].

**Table 1:** Preparation conditions of the Fe_3_O_4_@mSiO_2_ samples, their texture properties and drug holding capacities

**Composite sample**	**Amount of used TEOS (mL)**	**Average shell thickness (nm)**	***a***_**BET**_ **(m2/g)**	**Pore radius** ***r***_**p**_ **(nm)**	**Amount of loaded drug (mg**_**ibu**_ **/g**_**sample**_ **)**
SG-1	0.05	15.0	363.2	1.08	678
SG-2	0.07	25.0	493.1	1.08	828
SG-3	0.10	40.0	543.0	1.08	954

**Fig. 6:**
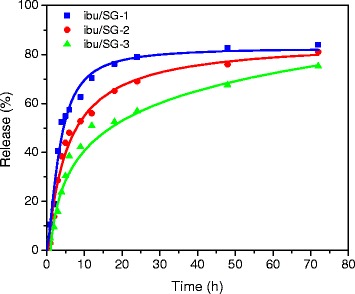
Release profiles of the ibuprofen loaded ibu/SG-1, ibu/SG-2, and ibu/SG-3 samples in SBF solution at 37 °C

Figure 6 shows the release profiles of the ibuprofen-loaded SG-1, SG-2, and SG-3 samples up to 72 hours in SBF solution. The amount of ibuprofen released in the SBF solution was estimated from the absorption spectra of the aliquots removed at different intervals. As can be seen, after 72 hours in SBF solution, about 81, 79, and 74 % of ibuprofen were released from the samples ibu/SG-1, ibu/SG-2, and ibu/SG-3, respectively. The release rate is considerably faster for the first 6 hours, as the drug incorporated at the porous surface of the nanostructures is released initially. The drug incorporated deeper inside the mesopores are released slowly, probably due to the strong capillary force acting on it. The contribution of capillary force on the release of ibuprofen drug is clear if we consider its release rate from the three samples during the initial 6 hours. The release rate is highest for the sample ibu/SG-1 with smaller silica shell thickness than the samples ibu/SG-2 and ibu/SG-3, which contain silica shells of higher thicknesses. The results indicate that the ibuprofen release rate can be controlled simply by controlling the thickness of the meso-silica layer around magnetite clusters.

**Fig. 7:**
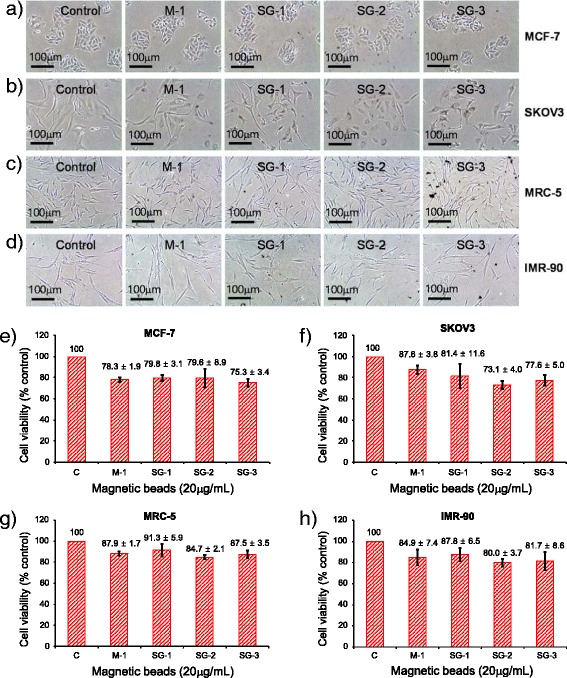
Comparison of cytotoxic effects of magnetic beads on human breast cancer cell line MCF-7, ovarian cancer cell line SKOV3, and normal human lung fibroblasts MRC-5 and IMR-90. Panels **a–d** are the phase-contrast microscopic images showing cell growth and colony morphology of MCF-7 (**a**), SKOV3 (**b**), MRC-5 (**c**), and IMR-90 (**d**) cell lines after magnetic beads treatments. Panels **e–h** show the cell viabilities of the used nanostructures in MCF-7, SKOV3, MRC-5, and IMR-90, respectively

To investigate the cytotoxicity of these magnetic nanostructures, the inhibitory potentials of M-1, SG-1, SG-2, and SG-3 samples were analyzed by viable cell counting. As shown in Fig. 7, these magnetic nanostructures have limited effects on the cell morphology and viability on human breast cancer line after incubation for 2 days at a concentration of 20 μg/mL. After 48 hours of treatment, samples M-1, SG-1, and SG-2 caused similar cytotoxicity (~20 %) in MCF-7 cells. Cytotoxicity induced by SG-3 sample was slightly higher (~25 %) than those of M-1, SG-1, and SG-2 samples (Fig. 7a, e). To test for cell-line-specific effects, we performed the same experiment in human ovarian cancer line SKOV3 (Fig. 7b, f) and in two normal human lung fibroblasts MRC-5 (Fig. 7c, g) and IMR-90 (Fig. 7d, h). The obtained results indicate that the cytotoxicity of the meso-silica-covered magnetite samples in normal human fibroblasts is a bit lower than that in probed cancer cell lines. Thus, our cytotoxicity tests through evaluation of cellular morphology and cell growth kinetics revealed that on covering with meso-silica layer, although the cytotoxicity of magnetite nanoparticles increases a bit on cancer cell lines, it does not change for normal human lung fibroblasts.

## Conclusions

In summary, we demonstrate the synthesis of Fe_3_O_4_@mSiO_2_ core-shell type nanostructures of high surface area (363, 493, and 543 m^2^/g) and ~2.16 nm of pore size, with acceptable biocompatibility (~80 %) for drug delivery applications. The nanostructures can hold medicinal drug such as ibuprofen as high as 954 mg/g_sample_, and present a good release behavior up to 81 % of the loaded drug. Covering magnetite nanoparticles by meso-silica layers protects them from body fluids without affecting their biocompatibility. Reasonable biocompatibility and good drug release performance of the nanostructures can be exploited for using them for targeted cancer and non-cancer drug delivery applications in human body.

## Additional file

Additional file 1: Figure S1. XRD patterns of the magnetite clusters prepared through hydrothermal method. Figure S2 FT-IR spectra of (a) Fe_3_O_4_@mSiO_2_ nanostructures (SG-3) before the acetic acid treatment and (b) SG-1, (c) SG-2, (d) SG-3 after their acetic acid treatment. Figure S3 Typical high-resolution SEM image of the magnetite clusters prepared by the hydrothermal method. Figure S4 a) UV absorption spectra of ibuprofen/hexane solutions with different ibuprofen concentrations used for preparing. b) Concentration calibration profile used for estimating drug loading in Fe_3_O_4_@mSiO_2_ composite nanoclusters.
