# Supplementary material for: Fabrication of Fe3O4@mSiO2 Core-Shell Composite Nanoparticles for Drug Delivery Applications
Source: Nanoscale Res Lett. 2015 May 13;10:217. doi: 10.1186/s11671-015-0920-5 (PMC4444644; doi:10.1186/s11671-015-0920-5)
Supplement: Additional file 1: Figure S1. — XRD patterns of the magnetite clusters prepared through hydrothermal method. Figure S2 FT-IR spectra of (a) Fe3O4@mSiO2 nanostructures (SG-3) before the acetic acid treatment and (b) SG-1, (c) SG-2, (d) SG-3 after their acetic acid treatment. Figure S3 Typical high-resolution SEM image of the magnetite clusters prepared by the hydrothermal method. Figure S4 a) UV absorption spectra of ibuprofen/hexane solutions with different ibuprofen concentrations used for preparing. b) Concentration calibration profile used for estimating drug loading in Fe3O4@mSiO2 composite nanoclusters. [file 11671_2015_920_MOESM1_ESM.docx]

***SUPPORTING INFORMATION***

Fabrication of Biocompatible Fe_3_O_4_@mSiO_2_ Core-shell Composite Nanoparticles for Drug Delivery Applications

Sergio Isaac Uribe Madrid, Umapada Pal*, Young Soo Kang, Junghoon Kim, Hyungjin Kwon, and Jungho Kim

Figure S1. XRD patterns of the magnetite clusters prepared through hydrothermal method.

Figure S1 shows the XRD pattern of Fe_3_O_4_ clusters prepared through hydrothermal method. The samples revealed diffraction peaks associated to magnetite in cubic phase (spinel inverse) (JCPDS #89-0691). The average crystallite size in the clusters was estimated using the Debye-Scherrer equation (*D* = 0.9* */*β *cos* *) on the most prominent (311) reflection of the sample. The estimated average crystallite size of the sample was 20 nm.

Figure S2. FT-IR spectra of (a) Fe_3_O_4_@mSiO_2_ nanostructures (SG-3) before the acetic acid treatment, and (b) SG-1, (c) SG-2, (d) SG-3 after their acetic acid treatment.

Figure S2 shows the FT-IR spectra of the Fe_3_O_4_@mSiO_2_ nanoparticles of different meso-silica layer thickness. As can be seen, apart from the absorption bands correspond to Fe-O bond of magnetite (580 cm^-1^), Si-O-Si (1090, 1200 cm^-1^), Si-O-H (961 cm^-1^), and Si-O (802 cm^-1^) bonds of silica shell, and O-H bond (1630 & 3430 cm^-1^) of surface adsorbed water, there appeared two absorption bands around 2921 and 2851 cm^-1^ for the untreated SG-3 sample (spectrum a, black line), which correspond to the -CH_2_- bond associated to CTAB present in the sample.^1^ For the acetic acid treated SG-1, SG-2, and SG-3 samples (green, blue, and red lines, respectively), these bands (2921 and 2851 cm^-1^) disappeared completely, indicating a complete removal of CTAB from the porous silica channels. The bands at 1090 and 961 cm^-1^ correspond to the asymmetric vibration of Si-O and Si-OH bonds, respectively. The shoulder peak at about 1200 cm^-1^ corresponds to asymmetric vibration of Si-O bond. The absorption band appeared at about 800 cm^-1^ corresponds to the symmetric vibration of Si-O bond. The absorption bands appeared around 3430 cm^-1^ and 1630 cm^-1^ correspond to the stretching and bending vibration modes of water absorbed at the surface of the nanostructures.^2,3^ Finally, the peak appeared around 580 cm^-1^ corresponds to the deformation of Fe-O bonds of magnetite.^4^ The FT-IR spectra of the samples confirm the presence of silica shells over the core magnetite particles as we claimed.


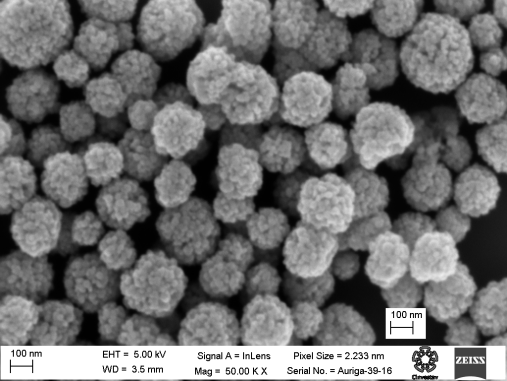


**Figure S3**. Typical high-resolution SEM image of the magnetite clusters prepared by the hydrothermal method.

The typical high-resolution SEM image presented in figure S3 shows the magnetite clusters consist of smaller nanoparticles (primary nanoparticles) of about 20 nm size.

b)

a)

**Figure S4**. a) UV absorption spectra of ibuprofen/hexane solutions with different ibuprofen concentrations used for preparing b) concentration calibration profile used for estimating drug loading in Fe_3_O_4_@mSiO_2_ composite nanoclusters.

Figure S4 (a) presents the UV-Vis absorption spectra of ibuprofen/hexane solutions with different ibuprofen concentrations. Figure S4 (b) shows the concentration calibration profile of ibuprofen prepared using the data of figure S4 (a) (considering the intensity of 263 nm absorption band).

REFERENCES

1 X. Fang, C. Chen, Z. Liu, P. Liu, and N. Zheng, *Nanoscale,* 2011, **3**, 1632.

2 K. H. S. Kung, and K. F. Hayes, *Langmuir,* 1993, **9**, 263.

3 A. Beganskienė, V. Sirutkaitis, M. Kurtinaitienė, R. Juškėnas, and A. Kareiva, *Material science*, 2004, **10**, 287.

4 M. Nikolić, K. P. Giannakopoulos, and V. V. Srdić, *Processing and Application of Ceramics,* 2010, **4**, 81.
